# Stable polyethylene glycol/biochar composite as a cost-effective photothermal absorber for 24 hours of steam and electricity cogeneration

**DOI:** 10.1039/d3ra06028d

**Published:** 2023-10-24

**Authors:** Belal N. Basuny, Doaa A. Kospa, Amr Awad Ibrahim, Ahmed Gebreil

**Affiliations:** a Department of Chemistry, Faculty of Science, Mansoura University Al-Mansoura 35516 Egypt amr_awad@mans.edu.eg; b Nile Higher Institutes of Engineering and Technology El-Mansoura Egypt

## Abstract

Seawater desalination powered by solar energy is the most environmentally and economical solution in responding to the global water and energy crisis. However, solar desalination has been negatively impacted by intermittent sun radiation that alternates between day and night. In this study, sugarcane bagasse (SCB) was recycled *via* the pyrolysis process to biochar as a cost-effective solar absorber. Besides, polyethylene glycol (PEG) as a phase change material was encapsulated in the abundant pore structure of biochar to store the thermal energy for 24 hours of continuous steam generation. The BDB/1.5 PEG evaporator exhibited an evaporation rate of 2.11 kg m^−2^ h^−1^ (98.1% efficiency) under 1 sun irradiation. Additionally, the BDB/1.5 PEG evaporator incorporated by the TEC1-12706 module for continuous steam and electricity generation with a power density of 320.41 mW m^−2^. Moreover, 10 continuous hours of evaporation were applied to the composite demonstrating outstanding stability. The composite exhibited high water purification efficiency through solar desalination due to the abundant functional groups on the biochar surface. Finally, the resulting low-cost and highly efficient PCM-based absorber can be used on a wide scale to produce fresh water and energy.

## Abstract 2

Seawater desalination powered by solar energy is the most environmentally and economical solution in responding to the global water and energy crisis.

## Graphical abstract

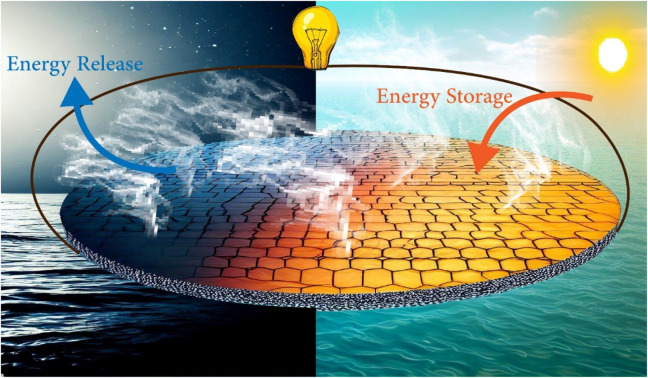

## 1. Introduction

The world's rising human population, along with the exploitation of water resources for home, industrial, and agricultural purposes, has resulted in a global scarcity of freshwater supply in many locations.^1^ Numerous viable solutions to this global problem have been proposed, including rainwater collecting, water recycling, and seawater desalination.^2,3^ As most of the accessible water resources are useless saline water, seawater desalination is a realistic way for freshwater production.^3^ A variety of desalination techniques were utilized including chemical filtration, multi-stage flash distillation, multiple-effect distillation, capacitive deionization, electro-dialysis, reverse osmosis (RO), and desalination batteries.^4^ However, the high cost, tremendous energy consumption, and secondary pollutants limit their wider commercialization.^5–7^ Thus, many studies have been conducted over the past several years on the utilization of solar energy to accomplish the evaporation of saline water and produce drinkable water.^8^ The solar desalination technique has the benefit of causing almost little environmental harm and using the least amount of energy possible.^9–11^

Solar energy, as clean and renewable energy, is a feasible solution to these challenges since it can both create purified water from seawater and support the development of ecologically friendly technologies.^12^ Solar steam generation (SSG) is one of the solar energy applications that has recently gained prominence due to its enormous potential for solar desalination or the treatment of industrial wastewater.^13^ When compared to ordinary water evaporation, SSG systems offer a much higher energy conversion efficiency.^4^ The recent efforts were focused on the improvement of water evaporation efficiency by reducing thermal losses and applying the majority of heat to the water phase change process.^14–16^ The most common SSG devices are usually made up of a photothermal layer (top layer), which is characterized by broadband light absorption and strong photothermal conversion and receiving quick and consistent water supply and a supporting layer (bottom layer) with a water channel and a low heat conductivity.^14,17–19^ The features of photothermal absorbers are critical to the SSG performance where the ideal absorber should have broadband solar absorbance, good hydrophilicity, porous networks, and superior efficiency of solar-to-heat conversion.^20,21^ Recently, several photoabsorbers have been exploited such as semiconductor materials,^22–26^ plasmonic nanometals (*e.g.*, Au, Ag, Al),^27,28^ ceramic-based materials,^29^ biomass-based materials,^30^ metal–organic frameworks (MOFs),^31^ covalent-organic frameworks,^32^ polymer-based materials,^33,34^ and carbon-based materials.^35,36^

Among all these, materials derived from biomass wastes have developed to be a substantial supply of carbon-based photothermal absorbers due to their low cost, simplicity of modification, and environmental friendliness.^37^ For the use of biomass materials for SSG, two basic techniques have been investigated including their utilization in pristine condition with no modifications to their structure and after their surface modifications by carbonization, polymer deposition, plasmolysis, or plasmonic metal decorating.^30^ However, these materials exhibit low evaporation rates, which reduces the effectiveness of collecting light and converting it to heat.^38^ To improve their light absorption capabilities, the biomass wastes could be converted into carbon-based material which is potentially more cost-effective than their disposal. One of the most significant commercial crops in many tropical and subtropical countries today is sugarcane. Bagasse is the sugarcane waste that is produced after the cane is extracted.^39^ Sugarcane bagasse (SCB) contains significant amounts of cellulose and hemicellulose, which can be depolymerized, carbonized, plasmolyzed, and modified by other processes.^40^ Some attention has been paid to the study and development of materials made from sugarcane.^41^ Biochar has been used in a range of energy and ecological applications, including energy storage and solar desalination.^42^ Pyrolysis, combustion, and hydrothermal processing are three ways to produce biochar.^43^ Pyrolysis at low temperatures is recommended to increase the efficiency of solar steam generation (SSG) because it maintains the abundant microchannels across the sugarcane bagasse structure, which increases the flow of water to the evaporator surface and helps to improve light scattering.^17,44^ Moreover, the microchannels of the biochar structure can allow the salt to return to the water improving the salt rejection ability.^45^ The additional advantage is the existence of different functional groups that have a high affinity for heavy metals and prevent salt formation on the surface.^46^

Solar desalination has been negatively impacted by intermittent sun radiation that alternates between day and night.^2^ Therefore, the incorporation of thermal energy storage materials into the photoabsorbers is a promising strategy to supply the heat for 24 hour desalination. Phase change materials (PCMs), also known as latent heat-storage materials, are a class of functional materials capable of storing and releasing enormous amounts of heat energy with little or no temperature change.^2,47^ PCMs have been developed for several applications including solar cooling and solar power plants, the space industry, photovoltaic electricity systems, waste heat recovery systems, and thermal energy storage.^48^ As PCMs for the thermal energy storage of a range of natural materials, paraffins, fatty acids, salt hydrates, and other compounds have all been used.^49^ Polyethylene glycol (PEG), as a thermal energy storage medium with a stable phase change, is a macromolecule that possesses advantageous features such as non-toxicity, good biocompatibility, biodegradability, hydrophilicity, and ease of chemical modification.^50,51^ The right phase transition temperature, high latent heat capacity, lack of supercooling, and low vapor pressure of PEG have also attracted a lot of attention.^52^ The main focus of scientific study at this time has been PCMs with good supporting materials that can maintain their structure even when the temperature exceeds the PCM's melting range.^53,54^ However, the leakage of PCMs during phase transition remains a significant barrier to their practical application.^55^ The most effective solution to this problem is the encapsulation of PCMs in an organic or inorganic matrix to produce shape-stable PCM composites.^56^ Many supporting materials have been used as matrices such as carbon nanotubes,^57^ montmorillonite nanosheets,^58^ mesoporous silica,^59^ graphene,^60^*etc.* The biochar can efficiently encapsulate PCMs due to its abundant pore structure.^55^

In this study, the sugarcane bagasse was recycled to biochar-derived bagasse (BDB) by thermal pyrolysis at low temperatures (350 °C) to keep the aligned microchannels *via* the biochar structure. The derived biochar was used as an efficient photothermal absorber due to its abundant carbon content, developed specific surface area, high porosity, high solar absorptivity, and abundant aligned microchannels which increases the water transportation to the surface. Moreover, the PEG with different ratios was encapsulated in the pore structure of the biochar to prevent the leakage of the PEG in the BDB/PEG composites. The BDB/1.5 PEG composite exhibited a high evaporation rate of 2.49 kg m^−2^ h^−1^ with a maximum light-to-heat conversion of 100%. The membrane exhibited high salt rejection owing to the biochar microchannels and functional groups which can bind the metal ions of the brine. An amount of heat dissipated to the bulk water through the SSG process and the recovery of this heat to thermoelectricity generation is the efficient strategy to utilize solar heat. According to the *Seebeck* effect, the temperature difference between the evaporator surface and bulk water can be converted to electricity by coupling a thermoelectric (TE) module with the solar evaporator. The hybrid system of BDB/1.5 PEG composite and TE-module can simultaneously produce a steam and electricity generation of 2.11 kg m^−2^ h^−1^ and 320.41 mW m^−2^, respectively. Finally, the PCM content of the device can store a certain amount of the converted heat enabling the hybrid system device to sustain a certain steam and power generation after lighting off. This work can promote a promising strategy for developing a cost-effective evaporator for simultaneous freshwater and electricity generation for 24 hours.

## 2. Experimental

### 2.1. Materials

Sugarcane bagasse collected from a nearby sugarcane juice shop (Mansoura, Egypt), Polyethylene glycol (PEG), ethanol (C_2_H_5_OH), sodium chloride (NaCl), polyurethane foam (PU-foam), potassium chloride (KCl), Magnesium sulfate (MgSO_4_·7H_2_O), Calcium nitrate (Ca(NO_3_)_2_), and DI water were used. The chemicals were utilized without further purification after being purchased from a significant chemical supplier with the highest purity grade.

### 2.2. Material synthesis

#### 2.2.1. Bagasse-derived biochar (BDB) synthesis

To begin, harvested sugarcane bagasse was chopped and processed using a crusher to a size of (2–3) mm pieces. The chopped sugarcane bagasse (CSB) was soaked in DI water and washed multiple times with ethanol and DI water to eliminate contaminants before being dried in an oven at 80 °C for 12 hours. The biochar synthesis was operated by the pyrolysis using a muffle furnace, which was elevated to 350 °C with a regulated heat rate of 5 °C min^−1^ and kept at that temperature for 2 hours.

#### 2.2.2. BDB/PEG composites synthesis

BDB/PEG composite was produced by using numerous concentrations (0.5, 1, and 1.5 mL) of polyethylene glycol (PEG) into a beaker with the dispersion of 0.5 g BDB which was ultrasonicated for 3 hours. For more than 24 hours and at 65 °C, the bio-composite solution was held under vacuum impregnation. With this technique, a unique shape-stable PEG/biochar structure was created.

### 2.3. Materials characterization

The composites were characterized using a variety of analytical methods. To demonstrate the existence of functional groups, FT-IR spectroscopy was performed using a MATTSON FT-IR-5000S spectrophotometer (resolution 4 cm^−1^). The crystal structure, crystallite size, and orientation of powder were investigated by X-ray diffraction (XRD) (Bruker, Cu–K radiation) at high angle 2° from (4 to 70) under (40) kV, step size of (0.02), *K*_α_ (1.54), 30 mA, and scan step time of 0.8 S. Using a scanning electron microscope equipped with a JEOL JSM 6510 lv and EDX techniques, SEM images of the PEG/BDB composites were produced. Besides, the materials' structure was determined by X-ray photoelectron spectroscopy (XPS) analysis utilizing a Thermo-Fisher Scientific ESCALAB 250 tool. To estimate the pore size distribution and the Brunauer–Emmett–Teller (BET) surface area of the adsorbents, the nitrogen adsorption/desorption measurements were performed using liquid nitrogen at 77 K. Meanwhile, the total pore volume was calculated using the adsorption of the nitrogen at a relative pressure of *P*/*P*^0^ = 0.99. Moreover, Alpha 300 Raman with a 532 nm argon ion laser was used to record the Raman spectra of the adsorbents from 1000 to 3000 cm^−1^. The samples were prepared for the measurements as follows; each as-synthesized adsorbent was dispersed in ethanol for 30 min, poured on the silicon wafer and then dried at an ambient temperature. Additionally, the elemental analysis of carbon, nitrogen, and hydrogen (CNH) was performed using an elemental analyzer equipped with a conductivity detector. The combustion and reduction tubes were assembled at 1150 and 850 °C, respectively, and the sulfanilamide was used as the CHN standard (N = 16.26%, C = 41.81%, S = 18.62%, and H = 4.65%, weight%). Moreover, a UV-Vis-NIR spectrometer from PerkinElmer was used to record the ultraviolet-visible-near-infrared (UV-Vis-NIR) spectra of materials to estimate the solar absorbance and reflectance of the samples. By heating the PEG/BDB composite sample to 800 °C at a heating rate of (10 °C min^−1^), thermal gravimetric analysis (TGA) may be used to assess the thermal characteristics of the materials. The heat flow and latent heat of a PEG/BDB composite sample that was enclosed in an aluminium pan and heated at a rate of (10 °C min^−1^) were assessed using differential scanning calorimeter (DSC) measurements. For the SSG performance, a 1 kW m^−2^ adjustable solar simulator outfitted with an AM 1.5G filter (a 250 W xenon light) was used. Throughout the seawater desalination, the temperature of the thermal composite absorber was measured using an infrared radiation (IR) (Hti-Xintai) camera and a thermocouple. A UV-Vis spectrophotometer was used to detect the amounts of contaminations including organic and inorganic before and after the SSG tests. The generated power was tested using a (TEC1-12705) module, and the current and potential were measured with a BT-METER (BT-90EPC Digital Multimeter) with a resistance of 5Ω.

### 2.4. Effectiveness of SSG under one sun

As a source of simulated solar light, a 300 watt xenon lamp with a 1-sun illumination setting was employed. The attempt to generate steam was conducted for a whole hour at a temperature of 25 °C. A 150 mL beaker with 100 mL DI water was used to support the SSG device. Every 5 minutes, a digital balance was used to record the water weight loss. The SSG evaporation devices typically consist of a photothermal layer (top layer), which is distinguished by broad-spectrum light absorption. It is obvious from its design that the supporting layer (bottom layer) is an insulator since it has a water channel.

The equation for calculating evaporation rate (*ν*):^13,14^

1 *ν* = d*m*/*S*d*t*

where *S* is the photothermal composite's surface area, *t* is the light exposure period, and *m* is the evaporation weight. Importantly, the system's evaporation rate at night was also monitored.

The equation for calculating energy efficiency (*η*):^13,14,61^

2 *η* = *H*_e_*v*/*Q*_in_

where *Q*_in_ is the density of the sunlight radiation (1 kW m^−2^) and *H*_e_ is the entire enthalpy of pure water's phase change that comes from the equation *H*_e_ = *C*Δ*T* + Δ*h*, where water specific heat capacity (*C* = 4.18 J g^−1^ k^−1^), water temperature (Δ*T*, K), and water vaporization enthalpy (Δ*h* = 2394 kJ kg^−1^).^62^

### 2.5. Effectiveness of steam and electricity production

To test the electricity generation, a special device was developed consisting of the TE module (covered by filter paper) to ensure continuous transmission of water and inserted between PU foam and the evaporator composite. At the same time, the BT-METER detected the difference in current between both module sides, as well as the potential of produced power for the TE module without membrane and with the evaporator composite in the dark and under solar illumination. Finally, using the collected current, the power density of the produced electricity can be calculated as follows:^63^

3 *P* = *I*^2^*R*

## 3. Results and discussion

### 3.1. Materials characterization

**Fig. 1:**
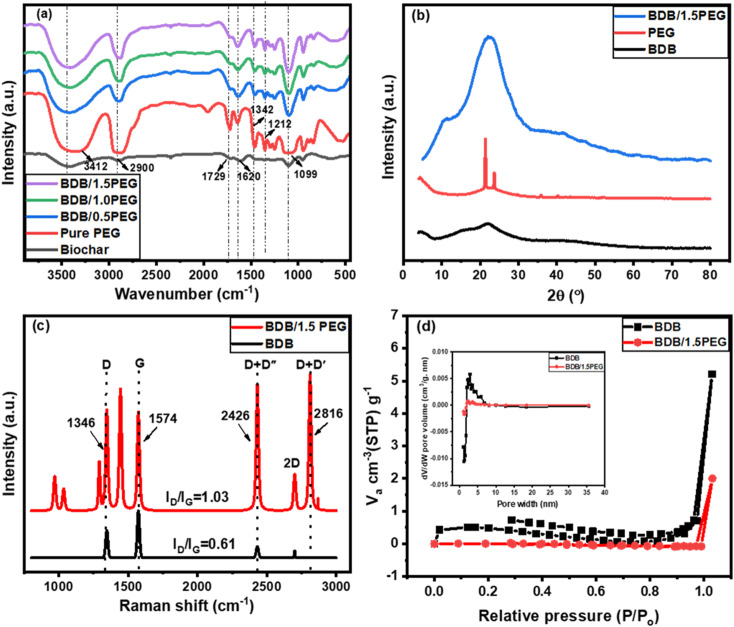
(a) Spectral data from the FT-IR for BDB, BDB/0.5 PEG, BDB/1.0 PEG, and BDB/1.5 PEG, (b) XRD of BDB, PEG and BDB/1.5 PEG, (c) Raman spectra of BDB and BDB/1.5 PEG, and (d) N_2_ adsorption–desorption isotherms of BDB and BDB/1.5 PEG (Insets showed the pore with distribution).

The FTIR spectra of BDB, pure PEG, BDB/0.5 PEG, BDB/PEG, and BDB/1.5 PEG were evaluated at a wavenumber range of 4000 to 500 cm^−1^ (Fig. 1a). For BDB, a large peak at 3412 cm^−1^ was observed in the FTIR spectra, which is caused by O–H bonds of the alcoholic and phenolic hydroxyl groups.^64^ At 2900 cm^−1^, the stretching vibrations of the unique C–H in the aliphatic group may be detected. The bonds of carboxylic groups or conjugated ketone are represented by the peak at 1729 cm^−1^, while the stretching vibrations of the aliphatic –C=C– appeared at 1620 cm^−1^.^64,65^ For pure PEG, the peak at approximately 3300 cm^−1^ is attributed to the stretching of the O–H groups of the polymer. Besides, the C–H stretching and bending vibrations of the methylene group were observed at approximately 2900 and 1467 cm^−1^, respectively. While the C–H bending and C–O stretching vibrations exhibited their characteristic bands that appear at 1342 and 1099 cm^−1^ are caused by, respectively; and the C–H twisting vibrations displayed a band at 1212 cm^−1^.^66–69^ For BDB/0.5 PEG, BDB/PEG, and BDB/1.5 PEG, the appearance of the peaks of biochar and PEG confirmed the incorporation of the biochar and PEG. Also, the small shift and the change in the intensity of the PEG peaks in the different composites indicated the strong interaction of the PEG and the biochar. Furthermore, when PEG concentration increases, the strength of the alkyl chain characteristic peak (2900 and 1476 cm^−1^) increases. To determine polymer crystalline structure and polymer orientations of photothermal absorbers, X-ray diffraction (XRD) was performed. The XRD spectra for BDB displayed the characteristic peaks of cellulosic structure at 2*θ* = 15.5, and 21.7° which were indexed as 101 and 002 miller indices, respectively.^17,70^ These crystallographic planes are the typical feature of amorphous carbon, revealing the amorphous properties of biochar.^71^ For PEG, the spectrum showed the two characteristic peaks at the 2*θ* of 21.2° and 23.6° representing the regular crystal structure of PEG.^55^ Moreover, the spectrum of BDB/1.5 PEG showed an overlapping between the characteristic peaks of biochar and PEG giving a broad peak with high intensity from 2*θ* of 14.7° to 2*θ* of 30.4°. Additionally, the broadness of this peak indicated the amorphous structure of the composite due to the high component of the amorphous biochar.

Additionally, the ordered and disordered structure of carbon in the biochar and BDB/1.5 PEG were depicted using Raman spectroscopy and represented in Fig. 1c and d. For the biochar spectrum, there are four characteristic peaks at 1341, 1574, 2433, and 2704 cm^−1^ attributed to the D, G, D + D′′, and 2D bands, respectively.^72^ The D band is due to the crystallite edge effect and the layer defects of materials. The G band is ascribed to band scattering of the sp^2^ carbon atoms' E_2g_ phonon, whilst the stacking order of nanosheets produces the 2D band which is an overtone of the D band.^73^ Generally, the ratio between the intensity of D and G peaks (*I*_D_/*I*_G_) estimates the disorder degree of carbon nanomaterials.^74^ It was noted that the defect degree of the carbon increases with the increase of the *I*_D_/*I*_G_ ratios. The ratio was calculated for the biochar (Fig. 1d) and was found to be 0.61 indicating the low disorder degree of the compound. On the other hand, the spectrum of the BDB/1.5 PEG showed the same characteristic peaks of the pure biochar with higher intensities in addition to a new peak at 2816 cm^−1^ related to the D + D′ band. The *I*_D_/*I*_G_ ratio of the composite increased from 0.61 for pure biochar to 1.03 for the composite indicating the increase in the disorder degree due to the incorporation of the PEG on the biochar surface. Furthermore, the adsorption and desorption of nitrogen gas can be performed for pure BDB and BDB/1.5 PEG composite to estimate the value of the BET surface area and the pore volume. Fig. 1d shows the adsorption–desorption isotherms which exhibit the type (II) curve with a low surface area of 3 m^2^ g^−1^ with a total pore volume of 0.003 cm^3^ g^−1^. The low specific surface area is due to the thermal treatment at low temperatures which kept the cellulosic structure of the biochar. After the incorporation of the PEG, the surface area and the pore volume of the biochar decreased to 1 m^2^ g^−1^ and 0.001 cm^3^ g^−1^ indicating the successful occupation of the biochar pores by PEG. Also, the average pore size of the pure BDB and BDB/1.5 PEG were found to be 6.4 and 1.2 nm, respectively (the inset of Fig. 1d).

**Fig. 2:**
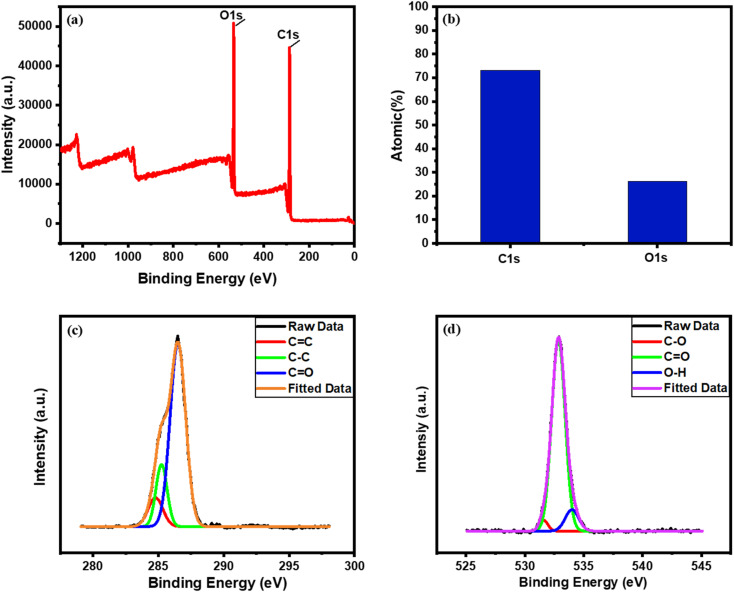
(a) Overall XPS spectral survey, (b) atomic percentage %, and (c and d) XPS spectrum of C1s and O1s of BDB/1.5 PEG, respectively.

The XPS spectra help to estimate the binding energy of the as-synthesized photo-thermal composite BDB/1.5 PEG which confirms the existence of C1s and O1s with the yield of 73.15% and 26.25%, respectively Fig. 2a and b. The C1s spectrum was displayed in Fig. 2c and revealed that the major surface functional groups were C–C, C=C, and C=O around 284.7, 285.2, and 286.5 eV, respectively.^17,75,76^ The O1's distinct peaks are depicted in Fig. 2d, which demonstrated that its main functional groups were O–C, O=C, and O–H at 531.5, 532.9, and 534 eV, respectively.^17,76^

**Fig. 3:**
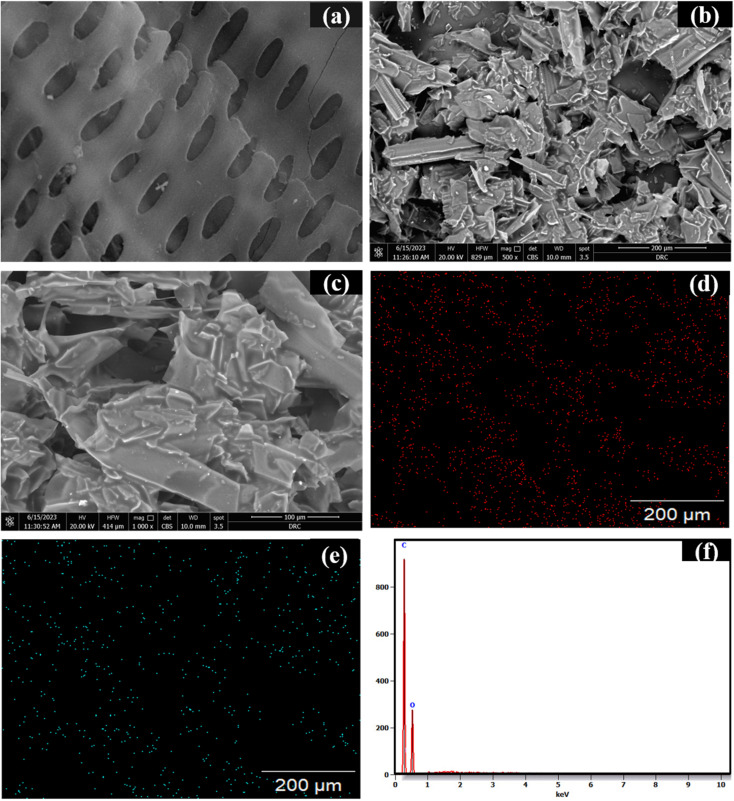
The photographs of SEM of (a) BDB, (b and c) BDB/1.5 PEG, (d) EDX mapping of C and (e) for O, and (f) EDX spectra of BDB/1.5 PEG.

The scanning electron microscope (SEM) is an effective method for examining and analyzing micro and nanoparticle imaging characterization of solid materials. BDB surface morphology was seen in SEM images, where the majority of their macroscopic shape had been retained. The characteristics of the formation of pores that are cylindrical help in water transportation and the spherical structure of biodegradable biochar (BDB) promoted with 350 °C pyrolysis (Fig. 3a). The SEM of BDB/1.5 PEG showed that the polyethylene glycol was effectively carried and distributed over the BDB surface without damaging the porous structure (Fig. 3b and c).^77^ The elemental composition of the BDB/1.5 PEG composite was examined using EDX analysis, showing the existence of C and O components (Fig. 3d and e). The EDX mappings were displayed in Fig. 3f and checked that these components were distributed uniformly over the substrate. Moreover, the elemental analysis (CHN) was used to estimate the percentages of carbon, nitrogen and oxygen in pure biochar and BDB/1.5 PEG composite. As shown in Table 1, the biochar exhibited a high amount of carbon indicating the concentration of carbon on the material after the pyrolysis process. Moreover, after the incorporation of the PEG, the carbon amount increases due to the high content of carbon in the PEG.

**Table 1:** Results of elemental analysis of BDB and BDB/1.5 PEG

Material	C (% m m^−1^)	H (% m m^−1^)	N (% m m^−1^)
BDB	52.9	2.3	0
BDB/PEG	68.8	4.1	0

**Fig. 4:**
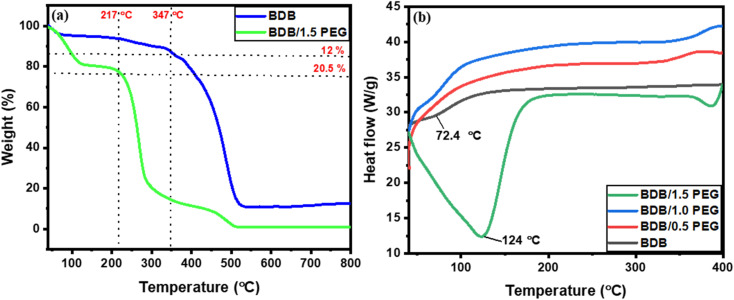
(a) TGA curves of BDB and BDB/1.5 PEG, and (b) DSC melting curves of BDB, BDB/0.5 PEG, BDB/PEG, and BDB/1.5 PEG.

Studying the thermal stability and weight loss % of BDB and BDB/1.5 PEG photo-thermal absorbers using thermogravimetric analysis (TGA) which was displayed in Fig. 4a. The TGA curve for BDB displayed that the weight loss was 12% around 350 °C, then reached the total weight loss of 100% at 525 °C. From the curve, the weight loss of BDB/1.5 PEG was 20.5% at a temperature of around 219 °C, revealing the composites' great stability until reached the total loss of 100% at 504 °C. Fig. 4b presented how the physical characteristics of a sample vary with temperature throughout time including phase change temperature using the melting DSC measurements. The melting endothermic peaks of BDB, BDB/0.5 PEG, BDB/1.0 PEG, and BDB/1.5 PEG composites were found to be 65.2, 67.4, 72.4, and 123.8 °C, respectively.

### 3.2. The performance of various synthesized thermal absorbers in terms of steam production

**Fig. 5:**
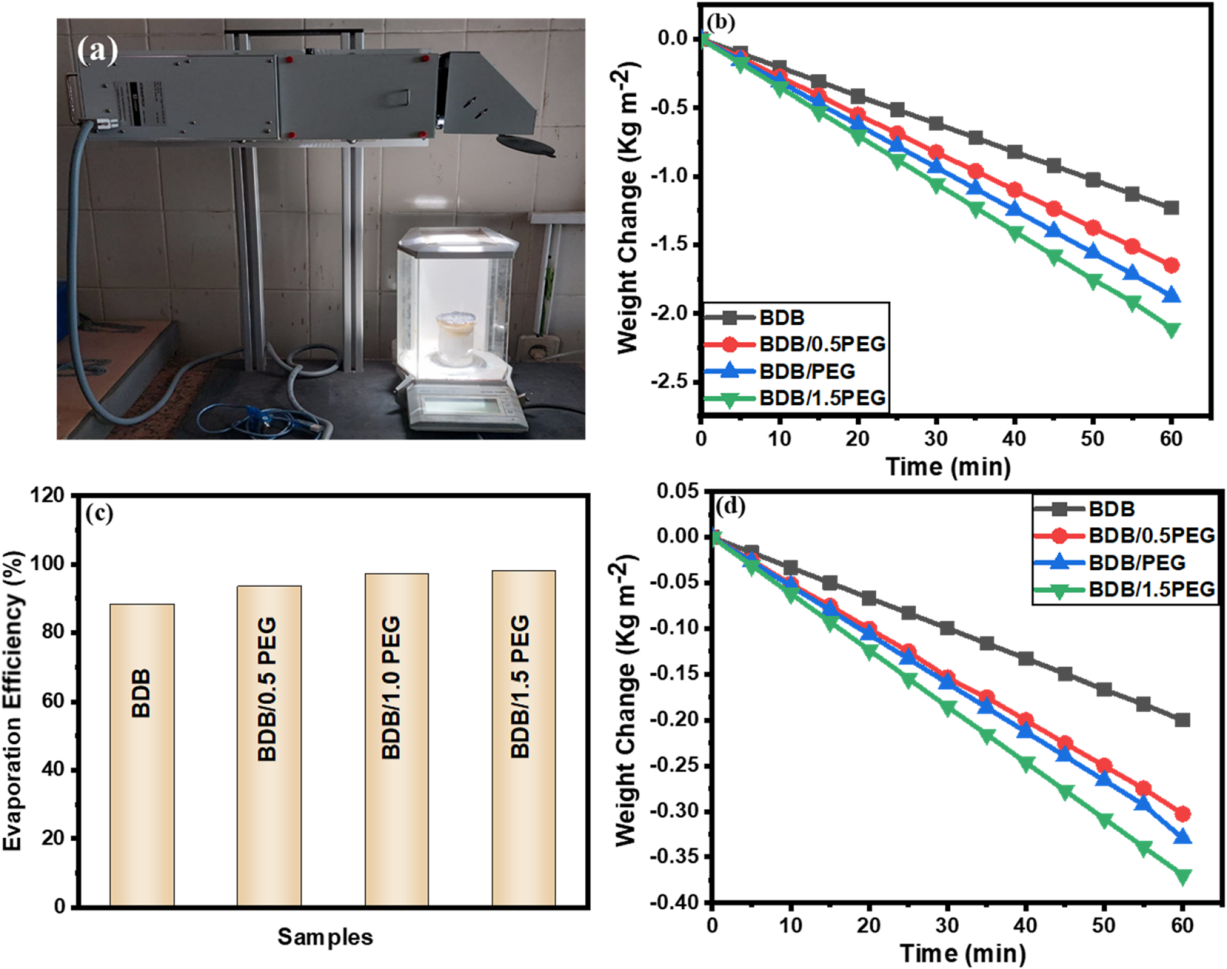
(a) picture of the SSG system under 1 sun lamp, (b) the weight change under 1 sun lamp, (c) the efficiencies curve of synthesized absorbers, and (d) the weight change when the lamp is off.

Four photo-thermal layers were created to evaluate the steam production effectiveness of the as-synthesized evaporators as mentioned: BDB, BDB/0.5 PEG, BDB/1.0 PEG, and BDB/1.5 PEG. As illustrated in Fig. 5, photo-thermal layers floating over DI water were subjected to 1 sun illumination for steam production testing. The SSG effectiveness of each sample was tested using a xenon light of 1 sun for 1 hour. Each sample was placed on top of a cylindrical piece of PU foam to allow the synthesized layer to float on the surface of 150 mL DI water while avoiding a shortage of solar heat. The foam was drilled, and the holes were filled with cotton to transport water to the surface. The weight change was monitored using a digital balance. The weight changes and rates of evaporation from the SSG production were mentioned for 60 min light on and off. BDB, BDB/0.5PEG, BDB/1.0PEG, and BDB/1.5 PEG absorbers at 1 sun illumination exhibited evaporation rates of 1.23, 1.65, 1.87, and 2.11 kg m^−2^ h^−1^, respectively (Fig. 5b). The addition of PEG as a phase change associated into the biochar capillaries enhanced the evaporation and led to the highest evaporation rate of 2.11 kg m^−2^ h^−1^. The obtained data was found to be comparable with that reported in the literature (Table 2). For BDB, BDB/0.5 PEG, BDB/PEG, and BDB/1.5 PEG, the estimated evaporation efficiencies of the as-synthesized layers were found to be 88.2%, 93.4%, 97.2%, and 98.1%, respectively Fig. 5c. The photothermal conversion mechanism occurred through three processes including absorption, scattering, and transmission. When the BDB/PEG is irradiated with the light that has the same energy, the photon interacts with the organic molecules (biochar and PEG) causing the electron excitation from the ground state to the excited state. Subsequently, the electrons relax to the ground state mainly through the nonradiative transition (*e.g.*, heat generation).^78^ On the other hand, when the light was turned off, BDB, BDB/0.5 PEG, BDB/PEG, and BDB/1.5 PEG absorbers produced evaporation of 0.2, 0.29, 0.28, and 0.36 kg m^−2^ h^−1^, respectively (Fig. 5d). The presence of PEG as thermal energy storage material can store an amount of the converted heat which enhanced the evaporation after the light off. Besides, the evaporation increased with the increase of PEG amount which increased the heat storage.

**Table 2:** Comparison of SSG performance of various polyacrylamide and biochar-based evaporators

Absorber	Support	Solar intensity (kW m^−2^)	Evaporation rate (kg m^−2^ h^−1^)	Efficiency (%)	References
Graphene oxide film	Polystyrene foam	1	1.45	94	79
Graphene networks (3DGNs)	Wood pieces	1	1.64	91.8	80
rGO/pDA-rGO membrane	PTFE	1	0.72	80	81
Carbonized layer of wood	Wood	1	1.04	75	82
Sponge-like hydrogel (LASH)	—	1	3.6	90	83
Ag/CuO-rGO	PU	1	2.6	92.5	13
Polyzwitterionic hydrogels	—	1	4.14	94	84
Cu/CuO foam	Lid	1	4.1	—	85
Paper-TREES	—	1	2.25	—	86
Ag/GO-PW@SiO_2_	PU	1	1.09	95.7	2
Biochar@PAAm	PU	1	2.71	98.7	38
1.5 PEG/BDB	PU	1	2.49	98.1%	This work

**Fig. 6:**
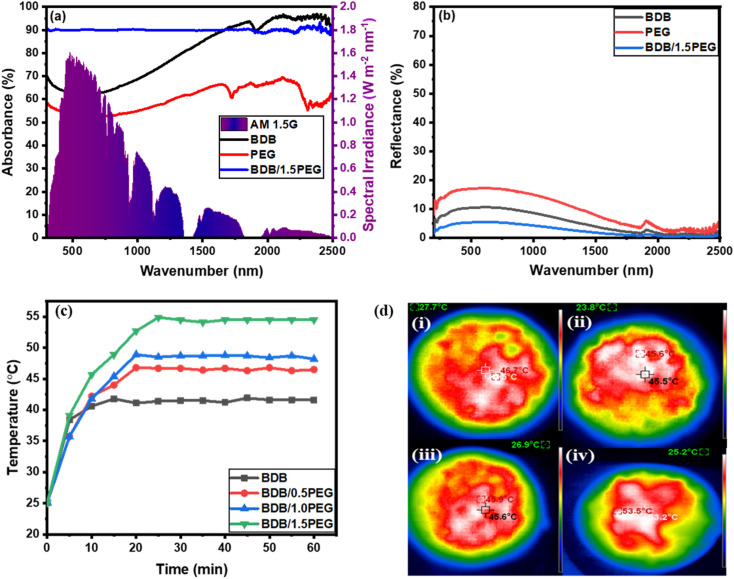
UV-Vis-NIR (a) absorbance spectra, (b) reflectance spectra of the as-prepared samples, (c) the temperature–time curve of the as-synthesized photothermal materials, and (d) infrared images of different absorbers (i) BDB, (ii) BDB/0.5 PEG, (iii) BDB/1.0 PEG, and (iv) BDB/1.5 PEG.

Additionally, the ideal evaporators should have strong solar absorbance and high solar-to-heat conversion. Therefore, the UV-Vis-NIR spectra of the prepared materials were recorded to estimate their absorbance over the solar wavelengths ranging from 300 to 2500 nm. The absorbance spectra of the samples were displayed in Fig. 6a and the reflectance was determined from Kirchhoff's law: *R* = 1 − (*T* + *A*).^38^ From Fig. 6a, the pure BDB and PEG exhibited high solar absorbance ranging from 50% to 90% with corresponding reflectance ranging from 10% to 25% (Fig. 6b) in the wavelength range of 400 to 1100 nm. The biochar and PEG exhibited high solar absorptivity owing to the carbon content of their structures. Moreover, the incorporation of the PEG and biochar exhibited high absorbance intensity (90%) with 4.5% reflectance owing to the high solar absorbance and storage of PEG and the microchannels of the biochar which increases the multiscattering of light in the sample.

After 1 hour of light exposure, the temperature variation at the surface of as-synthesized evaporators was detected using an infrared camera (IR) and a thermocouple. The temperatures at the photo-thermal composites' outer layer as a function of the temperature–time curve were recorded every 5 minutes for about 60 minutes under 1 sun illumination. The recorded temperature after 1 hour of light illumination was 46.6, 45.5, 46.2, and 53.7 °C for BDB, BDB/0.5 PEG, BDB/PEG, and BDB/1.5 PEG, respectively (Fig. 6c). It was observed that the temperature increased on the surface of BDB due to the high solar absorbance of the carbon structure of the biochar.^17^ Moreover, the temperature increased by the encapsulation of the PEG as a PCM material due to the high photothermal conversion efficiency of PEG.^87^ Also, the surface temperature of the membranes increased with the increase of the PEG amount. The maximum temperature of 53.7 °C after 60 min was reached by the BDB/1.5 PEG absorber indicating that it had the highest evaporation rate due to the combination of biochar and high amount of PEG. After 50 minutes of light, IR images of the evaporators were obtained, and the images matched the temperature–time curve (Fig. 6d).

### 3.3. Natural sunlight desalination

**Fig. 7:**
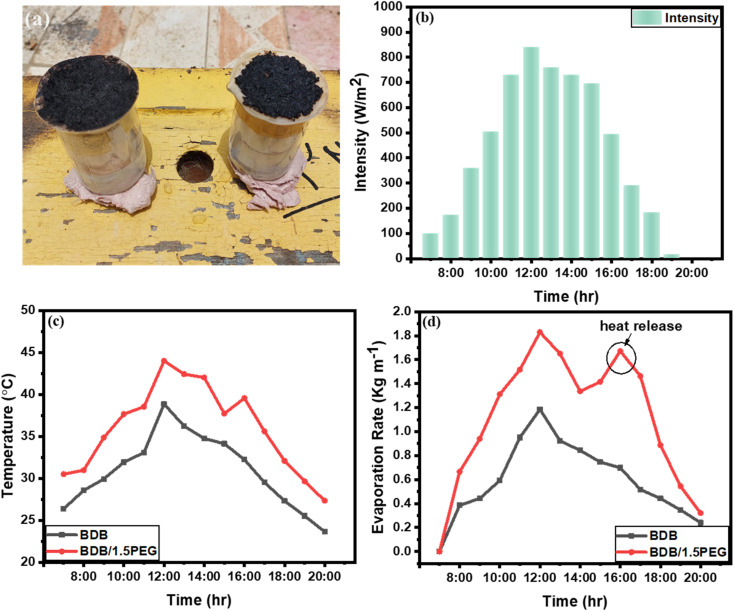
(a) Optical images of BDB and BDB/1.5 PEG composites under real sun, (b) intensity, (c) temperature, and (d) evaporation time curves of BDB and BDB/1.5 PEG composites under real sun.

For large-scale operation, the BDB and BDB/1.5 PEG evaporator was tested for 13 hours from 7 : 00 am to 8 : 00 pm to determine the significant potential (Fig. 7a). An electrical balance, thermo-couple, and power meter were used to record the weight loss, temperature change, and intensity of sunlight, respectively. The intensity of natural sunlight reached its maximum values of about 745 W m^−2^ at 12:00 pm (Fig. 7b). The temperature was recorded for BDB and BDB/1.5 PEG, and it was observed that their temperatures reached up to 30 and 35.4 °C, respectively (Fig. 7c). The total evaporation rate of BDB and BDB/1.5 PEG for 13 hours natural sunlight exposure was 9.33 and 10.12 kg m^−1^, respectively (Fig. 7d). The results were in agreement with the data under the solar simulator confirming that the PEG enhanced the solar absorptivity of the biochar. Also, it was observed that the evaporation rate at 7:00 pm increased using the BDB/1.5 PEG membrane compared to the BDB membrane due to the heat release from the PEG.

### 3.4. Effectiveness of SSG system and salt resistance

**Fig. 8:**
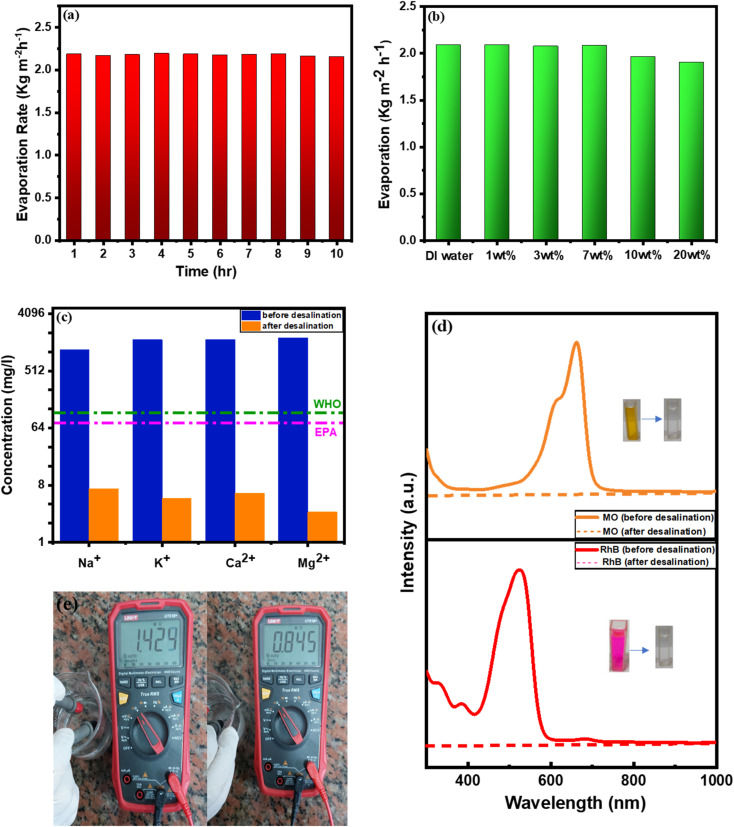
(a) 10 hours continuous evaporation curve of BDB/1.5 PEG composite, (b) BDB/1.5 PEG rate of evaporation curve for various water salinities, (c) metal-ion concentration, (d) dye ultraviolet-visible spectra previous to and following the SSG of BDB/1.5 PEG, and (e) resistance of water before and after desalination in ohm.

Additionally, 10 continuous hours of evaporation test were applied to the BDB/1.5 PEG composite. This test was carried out in seawater under 1 sun exposure to assess the reusability while recording weight loss every hour (Fig. 8a). The BDB/1.5 PEG composite showed comparable rates of evaporation values in both DI water and saline water when SSG was carried out in water with varying levels of sodium chloride (1%, 3%, 7%, 10%, and 20%), demonstrating the BDB/1.5 PEG composite's good salt rejection capability (Fig. 8b). ICP testing is used to detect and quantify a variety of chemical components required for metal sample analysis, analyzing the concentration of (K^+^, Mg^2+^, Na^+^, and Ca^2+^ ions) before and after evaporation during the process of desalination. The World Health Organization (WHO) established standard values for drinkable water, by comparing it with produced water, it was found that the BDB/1.5 PEG absorber has excellent ability for purification. It was discovered that the primary ion amount was radically reduced to low levels that are lower than those values (Fig. 8c). The effectiveness of a BDB/1.5 PEG absorber in the treatment of polluted water including (RhB and MO dyes) was tested. The UV spectra of water before and after solar evaporation demonstrated that the dyes have been completely removed from the generated water (Fig. 8d). Additionally, the resistance values were found to be 0.84 and 1.42 Ω before and after desalination, respectively (Fig. 8e), indicating the low amounts of conductive ions after desalination.

### 3.5. Performing continuous thermal electricity production

**Fig. 9:**
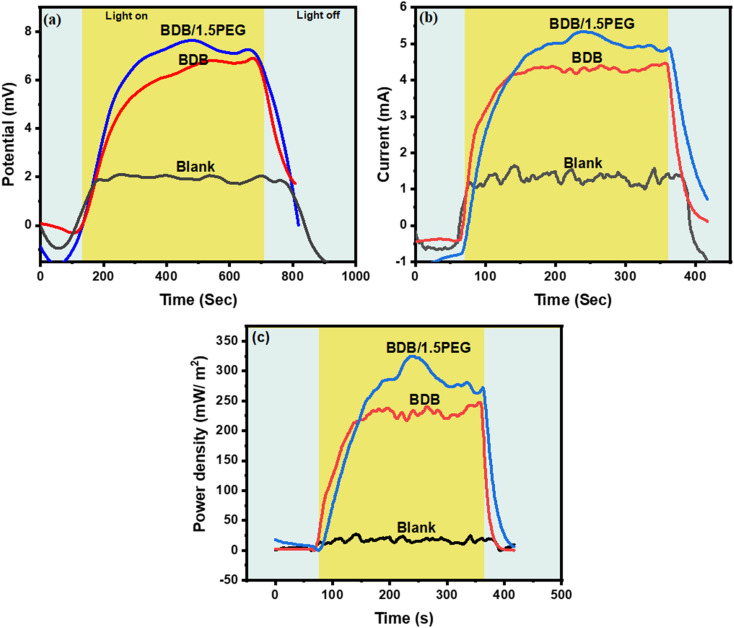
(a) current curve, (b) open-circuit voltage curve in ml volt, and (c) the equivalent output power density of a single TE module, BDB, and BDB/1.5 PEG under one sun.

The substantial variation in temperature between the water and the as-synthesized evaporator can be converted into electricity according to the *Seebeck* effect. The conversion can be performed by a thermoelectric module (TE-module) as follows; when one of each of the conductors gets heated, hot electrons move towards the cooled conductor producing electricity. The electricity generation process can be performed using a 10 Ω resistance and TEC1-12706 module with and without an evaporator. A TEC1-12706 module surrounded by filter paper for continuous movement of water through the solar steam generation. Open-circuit voltage has been determined in the dark as well as under one sun irradiation using a (BT-90EPC) device. Under one sun illumination it was recorded (2.06, 6.94, and 7.67 mV) for blank, biochar (BDB), and BDB/1.5 PEG, respectively (Fig. 9a). The induced current under 1 sun illumination for single module (blank), BDB, and BDB/1.5 PEG was (1.57, 4.51, and 5.37 mA), respectively (Fig. 9b). The power density was measured using the induced current and the internal resistance and found to be 27.4, 225, and 320.41 mW m^−2^ for module, BDB, and BDB/1.5 PEG, respectively (Fig. 9c).

## 4. Conclusion

In conclusion, we enhanced a 24 hour continuous steam-generating absorber using polyethylene glycol as a phase change material. The BDB/1.5 PEG composite exhibited a low coefficient of thermal conductivity, excellent light absorption, and storage, as well as it was chemically stable. The evaporation rate achieved by the BDB/1.5 PEG evaporator is about 2.11 kg m^−2^ h^−1^ and the power density was 320.41 mW m^−2^ under 1 sun irradiation, while the evaporation rate was 0.36 kg m^−2^ h^−1^ when light is off. The efficiency with dye-polluted water was eliminated, giving clear and pure water. The BDB/1.5 PEG composite showed great efficiency when SSG was carried out in water with varying levels of sodium chloride demonstrating that the composite had a good salt rejection capability. The resulting highly efficient PCM-based absorber may be utilized to generate fresh water and energy on a large scale.

## Conflicts of interest

There are no conflicts to declare.

## Supplementary Material

## References

[cit1] Humplik T., Lee J., O'Hern S. C., Fellman B. A., Baig M. A., Hassan S. F., Atieh M. A., Rahman F., Laoui T., Karnik R., Wang E. N. (2011). Nanostructured materials for water desalination. Nanotechnology.

[cit2] Younes Y. A., Kospa D. A., Salama R. S., Ahmed A. I., Ibrahim A. A. (2023). Hydrophilic candle wastes microcapsules as a thermal energy storage material for all-day steam and electricity cogeneration. Desalination.

[cit3] Zhang L., Jaroniec M. (2017). Toward designing semiconductor-semiconductor heterojunctions for photocatalytic applications. Appl. Surf. Sci..

[cit4] He J., Zhang Z., Xiao C., Liu F., Sun H., Zhu Z., Liang W., Li A. (2020). High-Performance Salt-Rejecting and Cost-Effective Superhydrophilic Porous Monolithic Polymer Foam for Solar Steam Generation. ACS Appl. Mater. Interfaces.

[cit5] Li W., Yang Y., Weber J. K., Zhang G., Zhou R. (2016). Tunable, Strain-Controlled Nanoporous MoS_2_ Filter for Water Desalination. ACS Nano.

[cit6] Neumann O., Urban A. S., Day J., Lal S., Nordlander P., Halas N. J. (2013). Solar vapor generation enabled by nanoparticles. ACS Nano.

[cit7] Tu T.-T., Lee M., Kuo S.-T., Den W. (2016). Citric acid-modified carbon chemical filtration for cleanroom air quality control: Study on N-methyl-2-pyrrolidone and the interference of co-existing toluene. Indoor Built Environ..

[cit8] Kospa D. A., Ahmed A. I., Samra S. E., El-Hakam S. A., Ibrahim A. A. (2022). Flexible CuO-rGO/PANI thermal absorber with high broadband photoresponse and salt resistance for efficient desalination of oil-contaminated seawater. Desalination.

[cit9] Chafik E. (2003). A new type of seawater desalination plants using solar energy. Desalination.

[cit10] Chafik E. (2003). A new seawater desalination process using solar energy. Desalination.

[cit11] Milow B., Zarza E. (1997). Advanced MED solar desalination plants. Configurations, costs, future—seven years of experience at the Plataforma Solar de Almeria (Spain). Desalination.

[cit12] Ghasemi H., Ni G., Marconnet A. M., Loomis J., Yerci S., Miljkovic N., Chen G. (2014). Solar steam generation by heat localization. Nat. Commun..

[cit13] Kospa D. A., Ahmed A. I., Samra S. E., Ibrahim A. A. (2021). High efficiency solar desalination and dye retention of plasmonic/reduced graphene oxide based copper oxide nanocomposites. RSC Adv..

[cit14] Shen C., Zhu Y., Xiao X., Xu X., Chen X., Xu G. (2020). Economical Salt-Resistant Superhydrophobic Photothermal Membrane for Highly Efficient and Stable Solar Desalination. ACS Appl. Mater. Interfaces.

[cit15] Zeng Y., Yao J., Horri B. A., Wang K., Wu Y., Li D., Wang H. (2011). Solar evaporation enhancement using floating light-absorbing magnetic particles. Energy Environ. Sci..

[cit16] Neumann O., Feronti C., Neumann A. D., Dong A., Schell K., Lu B., Kim E., Quinn M., Thompson S., Grady N., Nordlander P., Oden M., Halas N. J. (2013). Compact solar autoclave based on
steam generation using broadband light-harvesting nanoparticles. Proc. Natl. Acad. Sci. U.S.A..

[cit17] Saad A. G., Gebreil A., Kospa D. A., El-Hakam S. A., Ibrahim A. A. (2022). Integrated solar seawater desalination and power generation via plasmonic sawdust-derived biochar: Waste to wealth. Desalination.

[cit18] Li X., Li J., Lu J., Xu N., Chen C., Min X., Zhu B., Li H., Zhou L., Zhu S., Zhang T., Zhu J. (2018). Enhancement of interfacial solar vapor generation by environmental energy. Joule.

[cit19] Ma C., Yan J., Huang Y., Wang C., Yang G. (2018). The optical duality of tellurium nanoparticles for broadband solar energy harvesting and efficient photothermal conversion. Sci. Adv..

[cit20] Younes Y. A., Kospa D. A., Salama R. S., Ahmed A. I., Ibrahim A. A. (2023). Hydrophilic candle wastes microcapsules as a thermal energy storage material for all-day steam and electricity cogeneration. Desalination.

[cit21] Kospa D. A., Gebreil A., El-Hakam S. A., Ahmed A. I., Ibrahim A. A. (2023). Multifunctional plasmonic Ag–Cu alloy nanoparticles immobilized on reduced graphene oxide for simultaneous solar-driven steam, wastewater purification, and electricity generation. J. Mater. Res. Technol..

[cit22] Wang J., Li Y., Deng L., Wei N., Weng Y., Dong S., Qi D., Qiu J., Chen X., Wu T. (2017). High-Performance Photothermal Conversion of Narrow-Bandgap Ti_2_O_3_ Nanoparticles. Adv. Mater..

[cit23] Ding D., Huang W., Song C., Yan M., Guo C., Liu S. (2017). Non-stoichiometric MoO3-x quantum dots as a light-harvesting material for interfacial water evaporation. Chem. Commun..

[cit24] Yang B., Zhang Z., Liu P., Fu X., Wang J., Cao Y., Tang R., Du X., Chen W., Li S., Yan H., Li Z., Zhao X., Qin G., Chen X.-Q., Zuo L. (2023). Flatband λ-Ti_3_O_5_ towards extraordinary solar steam generation. Nature.

[cit25] Zhang X., Al-Musawi T. J., Alsaikhan F., Waleed I., Altimari U. S., Khaleel L. A., Hasan W. M. G., Koka N. A., Abosaooda M., Altamimi A. S., Cao Y. (2023). Effect of loading of Pt-decorated TiO_2_ on the enhancement of aerobic photo-oxidation of benzyl alcohol. Mol. Catal..

[cit26] Zhang Z.-Y., Li T., Yao J.-L., Xie T., Xiao Q. (2023). Mechanism and kinetic characteristics of photo-thermal dry reforming of methane on Pt/mesoporous-TiO_2_ catalyst. Mol. Catal..

[cit27] Zhou L., Tan Y., Wang J., Xu W., Yuan Y., Cai W., Zhu S., Zhu J. (2016). 3D self-assembly of aluminium nanoparticles for plasmon-enhanced solar desalination. Nat. Photonics.

[cit28] Bae K., Kang G., Cho S. K., Park W., Kim K., Padilla W. J. (2015). Flexible thin-film black gold membranes with ultrabroadband plasmonic nanofocusing for efficient solar vapour generation. Nat. Commun..

[cit29] Jonhson W., Xu X., Zhang D., Chua W. T., Tan Y. H., Liu X., Guan C., Tan X. H., Li Y., Herng T. S., Goh J. C.-H., Wang J., He H., Ding J. (2021). Fabrication of 3D-Printed Ceramic Structures for Portable Solar Desalination Devices. ACS Appl. Mater. Interfaces.

[cit30] Khajevand M., Azizian S., Boukherroub R. (2021). Naturally abundant green moss for highly efficient solar thermal generation of clean water. ACS Appl. Mater. Interfaces.

[cit31] Li G., Yue Q., Fu P., Wang K., Zhou Y., Wang J. (2023). Ionic dye based covalent organic frameworks for photothermal water evaporation. Adv. Funct. Mater..

[cit32] Ma F., Tang Q., Xi S., Li G., Chen T., Ling X., Lyu Y., Liu Y., Zhao X., Zhou Y., Wang J. (2023). Benzimidazole-based covalent organic framework embedding single-atom Pt sites for visible-light-driven photocatalytic hydrogen evolution. Chin. J. Catal..

[cit33] Guo Y., Zhao X., Zhao F., Jiao Z., Zhou X., Yu G. (2020). Tailoring surface wetting states for ultrafast solar-driven water evaporation. Energy Environ. Sci..

[cit34] Li W., Li Z., Bertelsmann K., Fan D. E. (2019). Portable Low-Pressure Solar Steaming-Collection Unisystem with Polypyrrole Origamis. Adv. Mater..

[cit35] He W., Zhou L., Wang M., Cao Y., Chen X., Hou X. (2021). Structure development of carbon-based solar-driven water evaporation systems. Sci. Bull..

[cit36] Abo El-Yazeed W. S., Abou El-Reash Y. G., Elatwy L. A., Ahmed A. I. (2020). Facile fabrication of bimetallic Fe–Mg MOF for the synthesis of xanthenes and removal of heavy metal ions. RSC Adv..

[cit37] Han J., Xing W., Yan J., Wen J., Liu Y., Wang Y., Wu Z., Tang L., Gao J. (2022). Stretchable and superhydrophilic polyaniline/halloysite decorated nanofiber composite evaporator for high efficiency seawater desalination. Adv. Fiber Mater..

[cit38] Tarek R., Kospa D. A., El-Hakam S. A., Ahmed A. I., Ibrahim A. A. (2023). Tailoring surface topography of biochar-based hydrogel for hazardous pollutants removal from contaminated seawater through simultaneous steam-electricity generation. Desalination.

[cit39] Inyang M., Gao B., Pullammanappallil P., Ding W., Zimmerman A. R. (2010). Biochar from anaerobically digested sugarcane bagasse. Bioresour. Technol..

[cit40] Chandel A. K., da Silva S. S., Carvalho W., Singh O. V. (2012). Sugarcane bagasse and leaves: foreseeable biomass of biofuel and bio-products. J. Chem. Technol. Biotechnol..

[cit41] Li Z., Lei S., Xi J., Ye D., Hu W., Song L., Hu Y., Cai W., Gui Z. (2021). Bio-based multifunctional carbon aerogels from sugarcane residue for organic solvents adsorption and solar-thermal-driven oil removal. Chem. Eng. J..

[cit42] Aup-Ngoen K., Noipitak M. (2020). Effect of carbon-rich biochar on mechanical properties of PLA-biochar composites. Sustainable Chem. Pharm..

[cit43] Yu S., Park J., Kim M., Ryu C., Park J. (2019). Characterization of biochar and byproducts from slow pyrolysis of hinoki cypress. Bioresour. Technol. Rep..

[cit44] Sadoun A. K., Gebreil A., Eltabey R. M., Kospa D. A., Ahmed A. I., Ibrahim A. A. (2022). Silver sulfide decorated carbonaceous sawdust/ES-PANI composites as salt-resistant solar steam generator. RSC Adv..

[cit45] Li J., Zhou X., Zhang J., Liu C., Wang F., Zhao Y., Sun H., Zhu Z., Liang W., Li A. (2020). Migration Crystallization Device Based on Biomass Photothermal Materials for Efficient Salt-Rejection Solar Steam Generation. ACS Appl. Energy Mater..

[cit46] Li H., Dong X., da Silva E. B., de Oliveira L. M., Chen Y., Ma L. Q. (2017). Mechanisms of metal sorption by biochars: Biochar characteristics and modifications. Chemosphere.

[cit47] Ismail K. A. R., Lino F. A. M., Machado P. L. O., Teggar M., Arıcı M., Alves T. A., Teles M. P. R. (2022). New potential applications of phase change materials: A review. J. Energy Storage.

[cit48] Meng Q., Hu J. (2008). A poly(ethylene glycol)-based smart phase change material. Sol. Energy Mater. Sol. Cells.

[cit49] Wang Y., Zhang Y., Xia T., Zhao W., Yang W. (2014). Effects of fabricated technology on particle size distribution and thermal properties of stearic–eicosanoic acid/polymethylmethacrylate nanocapsules. Sol. Energy Mater. Sol. Cells.

[cit50] Zhang L., Zhang P., Wang F., Kang M., Li R., Mou Y., Huang Y. (2016). Phase change materials based on polyethylene glycol supported by graphene-based mesoporous silica sheets. Appl. Therm. Eng..

[cit51] Sarı A., Alkan C., Biçer A. (2012). Synthesis and thermal properties of polystyrene-graft-PEG copolymers as new kinds of solid–solid phase change materials for thermal energy storage. Mater. Chem. Phys..

[cit52] Karaman S., Karaipekli A., Sarı A., Biçer A. (2011). Polyethylene glycol (PEG)/diatomite composite as a novel form-stable phase change material for thermal energy storage. Sol. Energy Mater. Sol. Cells.

[cit53] Qi G.-Q., Liang C.-L., Bao R.-Y., Liu Z.-Y., Yang W., Xie B.-H., Yang M.-B. (2014). Polyethylene glycol based shape-stabilized phase change material for thermal energy storage with ultra-low content of graphene oxide. Sol. Energy Mater. Sol. Cells.

[cit54] Zhong Y., Zhou M., Huang F., Lin T., Wan D. (2013). Effect of graphene aerogel on thermal behavior of phase change materials for thermal management. Sol. Energy Mater. Sol. Cells.

[cit55] Liu S., Peng S., Zhang B., Xue B., Yang Z., Wang S., Xu G. (2022). Effects of biochar pyrolysis temperature on thermal properties of polyethylene glycol/biochar composites as shape-stable biocomposite phase change materials. RSC Adv..

[cit56] Nazir H., Batool M., Bolivar Osorio F. J., Isaza-Ruiz M., Xu X., Vignarooban K., Phelan P., Inamuddin, Kannan A. M. (2019). Recent developments in phase change materials for energy storage applications: A review. Int. J. Heat Mass Transfer.

[cit57] Du X., Xu J., Deng S., Du Z., Cheng X., Wang H. (2019). Amino-Functionalized Single-Walled Carbon Nanotubes-Integrated Polyurethane Phase Change Composites with Superior Photothermal Conversion Efficiency and Thermal Conductivity. ACS Sustain. Chem. Eng..

[cit58] Yi H., Zhan W., Zhao Y., Qu S., Wang W., Chen P., Song S. (2019). A novel core-shell structural montmorillonite nanosheets/stearic acid composite PCM for great promotion of thermal energy storage properties. Sol. Energy Mater. Sol. Cells.

[cit59] Liu H., Niu J., Wang X., Wu D. (2019). Design and construction of mesoporous silica/n-eicosane phase-change nanocomposites for supercooling depression and heat transfer enhancement. Energy.

[cit60] Umair M. M., Zhang Y., Iqbal K., Zhang S., Tang B. (2019). Novel strategies and supporting materials applied to shape-stabilize organic phase change materials for thermal energy storage–A review. Appl. Energy.

[cit61] Zhao L., Du C., Zhou C., Sun S., Jia Y., Yuan J., Song G., Zhou X., Zhao Q., Yang S. (2020). Structurally Ordered AgNPs@C_3_N_4_/GO Membranes toward Solar-Driven Freshwater Generation. ACS Sustain. Chem. Eng..

[cit62] Chen Z., Li Q., Chen X. (2020). Porous Graphene/Polyimide Membrane with a Three-Dimensional Architecture for Rapid and Efficient Solar Desalination via Interfacial Evaporation. ACS Sustain. Chem. Eng..

[cit63] Liu X., Mishra D. D., Li Y., Gao L., Peng H., Zhang L., Hu C. (2021). Biomass-Derived Carbonaceous Materials with Multichannel Waterways for Solar-Driven Clean Water and Thermoelectric Power Generation. ACS Sustain. Chem. Eng..

[cit64] Elnour A. Y., Alghyamah A. A., Shaikh H. M., Poulose A. M., Al-Zahrani S. M., Anis A., Al-Wabel M. I. (2019). Effect of pyrolysis temperature on biochar microstructural evolution, physicochemical characteristics, and its influence on biochar/polypropylene composites. Appl. Sci..

[cit65] Zhao B., O'Connor D., Zhang J., Peng T., Shen Z., Tsang D. C. W., Hou D. (2018). Effect of pyrolysis temperature, heating rate, and residence time on rapeseed stem derived biochar. J. Cleaner Prod..

[cit66] León A., Reuquen P., Garín C., Segura R., Vargas P., Zapata P., Orihuela P. (2017). FTIR and Raman Characterization of TiO2 Nanoparticles Coated with Polyethylene Glycol as Carrier for 2-Methoxyestradiol. Appl. Sci..

[cit67] Devanand Venkatasubbu G., Ramasamy S., Ramakrishnan V., Kumar J. (2013). Folate targeted PEGylated titanium dioxide nanoparticles as a nanocarrier for targeted paclitaxel drug delivery. Adv. Powder Technol..

[cit68] Naghibi S., Madaah Hosseini H. R., Faghihi Sani M. A., Shokrgozar M. A., Mehrjoo M. (2014). Mortality response of folate receptor-activated, PEG-functionalized TiO_2_ nanoparticles for doxorubicin loading with and without ultraviolet irradiation. Ceram. Int..

[cit69] Abazović N. D., Comor M. I., Dramićanin M. D., Jovanović D. J., Ahrenkiel S. P., Nedeljković J. M. (2006). Photoluminescence of anatase and rutile TiO_2_ particles. J. Phys. Chem. B.

[cit70] Athira G., Bahurudeen A., Appari S. (2021). Thermochemical Conversion of Sugarcane Bagasse: Composition, Reaction Kinetics, and Characterisation of By-Products. Sugar Tech.

[cit71] Zhang Y., Cheng L., Ji Y. (2022). A novel amorphous porous biochar for adsorption of antibiotics: Adsorption mechanism analysis via experiment coupled with theoretical calculations. Chem. Eng. Res. Des..

[cit72] Huang K., Yu H., Xie M., Liu S., Wu F. (2019). Effects of poly(ethylene glycol)-grafted graphene on the electrical properties of poly(lactic acid) nanocomposites. RSC Adv..

[cit73] Fang M., Wang K., Lu H., Yang Y., Nutt S. (2010). Single-layer graphene nanosheets with controlled grafting of polymer chains. J. Mater. Chem..

[cit74] Ahmed A. I., Kospa D. A., Gamal S., Samra S. E., Salah A. A., El-Hakam S. A., Awad Ibrahim A. (2022). Fast and simple fabrication of reduced graphene oxide-zinc tungstate nanocomposite with enhanced photoresponse properties as a highly efficient indirect sunlight driven photocatalyst and antibacterial agent. J. Photochem. Photobiol., A.

[cit75] Jing F., Pan M., Chen J. (2018). Kinetic and isothermal adsorption-desorption of PAEs on biochars: effect of biomass feedstock, pyrolysis temperature, and mechanism implication of desorption hysteresis. Environ. Sci. Pollut. Res. Int..

[cit76] Jing S., Gong X., Ji S., Jia L., Pollet B. G., Yan S., Liang H. (2020). Self-standing heterostructured NiC_*x*_-NiFe-NC/biochar as a highly efficient cathode for lithium-oxygen batteries. Beilstein J. Nanotechnol..

[cit77] Barron M. K., Young T. J., Johnston K. P., Williams R. O. (2003). Investigation of processing parameters of spray freezing into liquid to prepare polyethylene glycol polymeric particles for drug delivery. AAPS PharmSciTech.

[cit78] Li J., Zhang W., Ji W., Wang J., Wang N., Wu W., Wu Q., Hou X., Hu W., Li L. (2021). Near infrared photothermal conversion materials: mechanism, preparation, and photothermal cancer therapy applications. J. Mater. Chem. B.

[cit79] Li X., Xu W., Tang M., Zhou L., Zhu B., Zhu S., Zhu J. (2016). Graphene oxide-based efficient and scalable solar desalination under one sun with a confined 2D water path. Proc. Natl. Acad. Sci. U.S.A..

[cit80] Kim K., Yu S., An C., Kim S.-W., Jang J.-H. (2018). Mesoporous Three-Dimensional Graphene Networks for Highly Efficient Solar Desalination under 1 sun Illumination. ACS Appl. Mater. Interfaces.

[cit81] Huang L., Pei J., Jiang H., Hu X. (2018). Water desalination under one sun using graphene-based material modified PTFE membrane. Desalination.

[cit82] Kuang Y., Chen C., He S., Hitz E. M., Wang Y., Gan W., Mi R., Hu L. (2019). A High-Performance Self-Regenerating Solar Evaporator for Continuous Water Desalination. Adv. Mater..

[cit83] Guo Y., Zhou X., Zhao F., Bae J., Rosenberger B., Yu G. (2019). Synergistic energy nanoconfinement and water activation in hydrogels for efficient solar water desalination. ACS Nano.

[cit84] Lei C., Guan W., Guo Y., Shi W., Wang Y., Johnston K. P., Yu G. (2022). Polyzwitterionic hydrogels for highly efficient high salinity solar desalination. Angew. Chem., Int. Ed..

[cit85] Liu X., Tian Y., Chen F., Caratenuto A., DeGiorgis J. A., Elsonbaty M., Wan Y., Ahlgren R., Zheng Y. (2021). An easy-to-fabricate 2.5D evaporator for efficient solar desalination. Adv. Funct. Mater..

[cit86] Bian Y., Ye Z., Zhao G., Tang K., Teng Y., Chen S., Zhao L., Yuan X., Zhu S., Ye J., Lu H., Yang Y., Fu L., Gu S. (2022). Enhanced Contactless Salt-Collecting Solar Desalination. ACS Appl. Mater. Interfaces.

[cit87] Yang H., Bai Y., Ge C., He L., Liang W., Zhang X. (2022). Polyethylene glycol-based phase change materials with high photothermal conversion efficiency and shape stability in an aqueous environment for solar water heater. Composites, Part A.

